# Assessment of Health-Related Behaviors in Patients Hospitalized with Chronic Psychiatric Disorders—A Case-Control Study from a Closed Psychiatric Ward

**DOI:** 10.3390/nu17142315

**Published:** 2025-07-14

**Authors:** Maciej Domański, Anna Domańska, Zuzanna Chęcińska-Maciejewska, Sabina Lachowicz-Wiśniewska, Wioletta Żukiewicz-Sobczak

**Affiliations:** 1 Department of Nutrition and Food, The Faculty of Medicine and Health Science, University of Kalisz, plac Wociecha Bogusławskiego 2, 62-800 Kalisz, Poland; maciej.domanski@interia.pl (M.D.); anna_domanska@onet.pl (A.D.); zuchecinska@gmail.com (Z.C.-M.); 2 Department of Biological Bases of Food and Feed Technologies, University of Life Science in Lublin, 13 Akademicka Street, 20-950 Lublin, Poland

**Keywords:** dementia, dietary intake, F03, F06.2, health behavior, psychiatric disorders

## Abstract

Background: Severe psychiatric disorders are frequently associated with disruptions in health-related behaviors, including diet and lifestyle. This cross-sectional study aimed to assess and compare selected dietary and lifestyle behaviors among long-term psychiatric inpatients diagnosed with unspecified dementia (F03) or organic delusional disorder (F06.2) and a control group of mentally healthy individuals. Methods: A 50-item validated questionnaire was administered to 28 hospitalized patients and 10 control participants. Analyses included nutritional habits, physical activity, stimulant use, and hydration, using non-parametric tests and effect size indicators (Cramér’s V). Results: Significant differences were observed in meal regularity, frequency of meals, types of beverages consumed, and physical activity. Strong associations were found for meal types (V = 0.590) and stress-induced eating (V = 0.525). Conclusions: The observed behavioral differences may reflect disease-related effects, demographic variation, or a combination of both. Despite these limitations, the findings suggest key areas for further investigation and support the need for targeted dietary and lifestyle interventions in psychiatric settings.

## 1. Introduction

Mental disorders represent a significant public health challenge worldwide. According to the World Health Organization, more than 450 million people suffer from various forms of mental disorders, and approximately 25% of the population experiences symptoms of poor mental health each year. In Poland, a systematic increase in the number of psychiatric hospitalizations has been observed, indicating the growing importance of this group of patients to the healthcare system. Mental disorders such as depression, schizophrenia, and anxiety disorders often coexist with unhealthy dietary habits, which can worsen patients’ conditions and hinder the treatment process [1,2,3,4].

An increasing body of research highlights a significant link between mental health and lifestyle, including diet. The Mediterranean diet, rich in vegetables, fruits, unsaturated fatty acids, fermented products, omega-3 fatty acids, and antioxidants, may exert a protective effect on the nervous system, whereas the Western diet, characterized by processed foods, simple sugars, and trans fats, increases the risk of depression and anxiety [5,6,7,8,9]. A diet rich in vegetables, fruits, whole grains, and healthy fats may support mental health, while one high in processed foods may deteriorate it. In recent years, there has been growing emphasis on the key role of the gut microbiota in regulating the function of the nervous system, immune system, and cortisol levels, particularly via the so-called gut–brain axis [7,10,11]. This axis includes bidirectional communication between the gastrointestinal tract, the central nervous system, and the immune system, as well as complex metabolism of intestinal microbes that influences neurotransmission, neuroinflammation, and neuroplasticity [12,13].

Diet plays a fundamental role in modulating the composition and activity of the microbiota. The intake of dietary fiber, fermented products, unsaturated fatty acids, and polyphenols promotes the growth of beneficial bacteria such as Lactobacillus and Bifidobacterium, which support the production of short-chain fatty acids (SCFAs) known for their anti-inflammatory and neuroprotective properties [14,15]. In contrast, a diet high in simple sugars, saturated fats, and ultra-processed foods can lead to dysbiosis—a disruption in microbial balance in the gut—associated with the intensification of depressive and anxiety symptoms, as well as a decline in cognitive function [16]. Among individuals with mental disorders, who often present with irregular meals, poor dietary quality, and a tendency toward “comfort” foods, the risk of dysbiosis may be particularly high [17]. Studies have shown that dietary interventions aimed at improving microbiota quality can reduce depressive symptoms and improve cognitive function, which opens new perspectives for supporting psychiatric therapy through appropriately tailored nutrition [18].

Therefore, maintaining proper dietary habits, including adequate intake of fiber and polyphenols and limiting processed foods, should be an essential component of comprehensive care for patients with mental disorders. Integrating dietary components into therapeutic strategies may improve not only somatic but also mental health, enhancing the effectiveness of pharmacological and psychotherapeutic treatment [19,20,21,22,23].

Despite the growing number of publications on mental health and nutrition, there remains a lack of studies focusing on the actual dietary behaviors of patients staying in psychiatric hospitals, especially in closed wards. The few studies addressing this population point to significant deficits in nutritional education and poor diet quality. Nutritional interventions are also lacking in psychiatric treatment, which constitutes a critical gap in care for patients with mental disorders [7], despite evidence from Fojcik et al. [8] indicating that health education can improve self-esteem, treatment engagement, and reduce disease symptoms [24,25,26,27].

The aim of this study was to evaluate selected health-related and dietary behaviors among long-term hospitalized psychiatric patients diagnosed with unspecified dementia (F03) and organic delusional disorder (F06.2) and to compare them with a control group of individuals without diagnosed mental illness. In addition to identifying the frequency of pro- and anti-health behaviors—such as regularity of meals, food product choices, physical activity, and stimulant use—the study also explored the influence of stress on eating behaviors and patients’ awareness of healthy lifestyle principles. This research attempts to address the gap in the current literature by focusing on individuals with chronic organic psychiatric disorders, a population for which dietary and lifestyle studies are limited. The results may support the development of targeted nutritional and educational strategies in psychiatric care and inform future intervention programs aimed at improving mental health through lifestyle modification.

## 2. Materials and Methods

This cross-sectional study aimed to compare selected health-related and dietary behaviors among long-term hospitalized psychiatric patients with a diagnosis of unspecified dementia (F03) or organic delusional disorder (F06.2) and a control group of individuals without diagnosed mental illness. The research focused on specific behaviors such as eating habits, food choices, physical activity, stimulant use, and the perceived impact of stress on diet and lifestyle.

The study was conducted between January and December 2022 in the closed psychiatric ward of the Neuropsychiatric Hospital in Lublin, Poland. Ethical approval was obtained from the Bioethics Committee of the Kalisz Academy (Resolution No. 5/2021), and all participants provided informed consent prior to participation.

A total of 28 inpatients from the psychiatric ward and 10 individuals from a general outpatient clinic (control group) were recruited. Participants in the clinical group had been hospitalized for a minimum of several months and were under continuous medical supervision, allowing the attending psychiatrist to assess cognitive capacity and determine eligibility for reliable interview participation. Control group members were individuals without a psychiatric diagnosis who agreed to complete the survey during routine medical visits.

### 2.1. Research Group

The inclusion criterion for the study group was a diagnosis of a mental disorder according to the ICD-10 classification: F03 (unspecified dementia) or F06.2 (organic delusional disorder). A total of 28 long-term inpatients (18 women and 10 men) were recruited from the Psychiatric Care and Treatment Facility (ZLOP) at the M. Kaczyński Neuropsychiatric Hospital in Lublin. Among them, 16 patients (12 women and 4 men) were diagnosed with F03, and 12 patients (6 women and 6 men) with F06.2.

Although these are distinct psychiatric diagnoses, both are classified as chronic psychiatric disorders under ICD-10 and share similar treatment environments. All patients received standardized psychiatric care, including pharmacotherapy and individually tailored dietary support, and were hospitalized in the same ward under consistent therapeutic supervision. Therefore, for the purposes of behavioral analysis, they were treated as a single observational group. Diagnoses were established according to ICD-10 criteria, which were in clinical use during the study period (January–December 2022).

### 2.2. Control Group

The control group consisted of individuals without psychiatric diagnoses who were recruited during routine outpatient visits and were available for voluntary participation under similar procedural conditions at the Primed Medical Centre in Lublin. Participants voluntarily consented to participate in the study in accordance with the approved protocol. The control group included 10 participants (8 women and 2 men).

All psychiatric patients included in the study were long-term inpatients from the same closed psychiatric ward. Despite the presence of dementia or organic affective disorders (ICD-10 codes F03 and F06.2), only individuals assessed by the attending psychiatrist as having sufficient cognitive capacity to understand and respond to the survey questions were included. This selection ensured basic reliability of self-reported data.

The control group consisted of adult patients attending outpatient visits to primary care clinics for general medical concerns, with no diagnosed psychiatric disorders. While the groups were not matched for age or education, the control group was selected based on clinical comparability and availability for voluntary participation.

### 2.3. Research Methods

The survey was conducted by the attending physician, a specialist in psychiatry, during the patients’ hospitalization at the M. Kaczyński Neuropsychiatric Hospital in Lublin. A custom-designed questionnaire was developed based on clinical dietetic experience and previous nutritional assessments. It was refined through pilot testing and practical application to ensure clarity, coherence, and thematic relevance. Although not formally standardized, the tool was internally validated for consistency and content completeness. The questionnaire consisted of 50 items covering demographics, dietary behaviors, physical activity, and stimulant use. Physical activity was defined in accordance with the World Health Organization (WHO) as any bodily movement produced by skeletal muscles that requires energy expenditure. For participants in the control group, the survey was administered at the Primed Medical Centre in Lublin, following qualification by a primary care physician (PCP) with specialization in psychiatry.

The “Dietary Habits and Physical Activity” questionnaire included (i) demographic data (9 items)—gender, age, place of residence, level of education, subjective financial status, perceived health status, subjective dietary evaluation, average sleep duration, and sources of information about a healthy lifestyle; (ii) nutritional behaviors (32 items)—meal quantity, quality, regularity, and timing; time of last meal; the influence of stress on food quality and quantity; appetite disturbances; dietary preferences and their impact on food choices; supervision of meals; use of institutional food services; and consumption of specific food categories including high-quality proteins, fermented foods, dairy products, spreads, beverages, vegetables, fruits, nuts, sweets, and ultra-processed foods; (iii) physical activity (6 items)—regularity, frequency, motivation, perceived benefits, and demotivating factors; (iv) stimulant use (3 items)—alcohol, drug use, and smoking.

This study is a continuation of previously published research titled Hematological Correlations as Predictors of Disease Manifestations in Psychiatric Inpatients [28]. The same study and control groups were used in both studies; therefore, the technical details regarding sample selection and methodological framework are presented in the cited publication.

The questionnaire was developed ad hoc by a multidisciplinary research team composed of a psychiatrist, a clinical dietitian, and a public health methodologist. It was designed specifically for this study to assess health-related behaviors among psychiatric inpatients. The tool included items on dietary habits, physical activity, substance use, and perceived health status. A pilot version was administered to 10 patients (not included in the final sample) to assess clarity, content coverage, and acceptability. Based on feedback, minor adjustments were made, and internal consistency was confirmed using Cronbach’s alpha (α = 0.81), indicating good reliability.

### 2.4. Statistica

All statistical analyses were performed using Statistica 13.3 software (StatSoft, Kraków, Poland). Descriptive statistics—including mean, standard deviation, minimum, maximum, and percentage—were used to characterize both the study and control groups. The chi-square (χ^2^) test was applied to assess associations between categorical variables, while the Kruskal–Wallis test was used for ordinal and non-normally distributed continuous variables. For two-group comparisons involving non-normally distributed data, the Mann–Whitney U test was employed. A *p*-value < 0.05 was considered statistically significant. The internal consistency of the custom-designed questionnaire was evaluated using Cronbach’s alpha coefficient, which yielded a value of α = 0.81, indicating good internal reliability. To assess the strength of association between group affiliation (psychiatric vs. control) and categorical variables such as dietary habits, meal regularity, or stress-related behaviors, Cramér’s V coefficient was calculated alongside the chi-square test. This statistic is suitable for nominal variables in contingency tables. A Cramér’s V value of <0.1 indicates a negligible relationship, 0.1–0.3 a weak relationship, 0.3–0.5 a moderate relationship, and >0.5 a strong relationship.

## 3. Results

### 3.1. Sociodemographic Characteristics

Significant differences were observed between the study and control groups in terms of age, education level, and place of residence. The psychiatric group was significantly older (*p* < 0.05), more likely to live in rural areas or smaller towns, and had lower levels of formal education.

In the study group (*n* = 28), 36% were female and 64% male, while in the control group (*n* = 10), 30% were female and 70% male. Age distribution in the study group was as follows: 54% aged 58–68 years, 11% aged 47–57, 29% aged 36–46, and 7% over 69. In the control group, 30% were aged 18–25, 40% aged 36–46, 20% aged 47–57, and 10% aged 58–68.

With regard to place of residence, 36% of patients in the study group lived in cities with over 100,000 inhabitants, 43% in smaller towns, and 21% in rural areas. In contrast, 80% of the control group lived in large cities and 20% in smaller towns. In terms of education, 29% of the study group had higher education (including 21% with a university degree), while 32% had basic or vocational education. In the control group, 90% had higher education, including 60% with a university degree, and only 10% had vocational training.

Regarding BMI, 68% of patients in the study group fell within the normal range (18.5–24.9 kg/m^2^), and 32% were overweight (25.0–29.9 kg/m^2^). In the control group, 50% had a BMI within the normal range, 40% were overweight, and 10% were obese (BMI ≥ 30). Descriptive data and statistical comparisons are presented in Table 1.

Although no statistically significant differences (*p* > 0.05) were found for some variables, Cramér’s V coefficients indicated moderate associations for age (V = 0.31) and education (V = 0.38), suggesting meaningful demographic divergence. A weaker association was observed for place of residence (V = 0.25), while gender (V = 0.00) and BMI (V = 0.00) showed no relevant differences. These results align with chi-square analyses and highlight selected sociodemographic disparities between the groups.

### 3.2. Pro-Health and Anti-Health Nutritional Behaviors

Patients in the psychiatric group exhibited less favorable nutritional behaviors compared to the control group (Table 2). They were significantly more likely to skip meals, consume fewer fresh vegetables and fruits, and rely more heavily on highly processed foods (*p* < 0.05). A notably lower proportion of psychiatric patients reported regular breakfast consumption. These patterns suggest poorer dietary habits in this clinical population. Moderate to strong associations were observed for skipping meals (Cramér’s V = 0.51) and the consumption of processed foods (Cramér’s V = 0.48), indicating considerable behavioral divergence between the study and control groups.

Additional differences emerged in subjective quality-of-life parameters. In terms of material condition, 36% of the psychiatric patients rated it as “neither good nor poor”, 29% as “poor”, 21% as “good”, 11% as “very good”, and 4% as “very poor”. In contrast, 70% of control group participants rated their condition as “good”, 20% as “very good”, and 10% as “neither poor nor good”. Regarding health status, 50% of patients with psychiatric disorders rated their health as “good”, 46% as “poor”, and 4% as “very poor”. In the control group, 70% rated their health as “good”, 20% as “very good”, and 10% as “poor”.

Self-assessment of nutritional habits showed that 93% of psychiatric patients considered their diet to be “good”, while 7% rated it as “poor”. In the control group, 80% assessed their diet as “good” and 20% as “very good”. Regarding sleep duration, 29% of psychiatric patients reported sleeping less than 7 h per day, 54% between 7 and 8 h, and 18% more than 8 h. Among controls, 10% reported <7 h of sleep, 80% between 7 and 8 h, and 10% > 8 h.

Differences also emerged in sources of health-related knowledge. Among psychiatric patients, 54% reported relying on television and radio, 21% on the internet, 21% on books and magazines, and 4% on family members. In the control group, 80% relied on the internet, while 20% used television and radio as primary sources of health information.

Statistical analysis revealed that these differences were significant for all parameters (*p* < 0.05), except for dietary self-assessment and sleep duration. Moderate associations were found for material condition (Cramér’s V = 0.31) and source of health information (V = 0.35), indicating that psychiatric patients more often experienced financial strain and relied on traditional media. A weaker association was observed for perceived health status (V = 0.28), suggesting a trend toward poorer self-evaluation among psychiatric patients. In contrast, sleep duration (V = 0.07) and dietary self-assessment (V = 0.00) showed negligible differences between the groups (Table 2).

These findings confirm that individuals hospitalized for chronic psychiatric conditions present with a distinct pattern of nutritional and lifestyle behaviors that may require targeted educational and dietary interventions in clinical settings.

### 3.3. Dietary Patterns and Lifestyle Behaviors

#### 3.3.1. Meal Frequency and Composition

The majority of patients in the psychiatric group (86%) consumed three meals per day, with 11% consuming four meals and 4% consuming two. In the control group, 70% consumed three meals, 20% four meals, and 10% five meals daily. All participants in both groups reported eating breakfast and supper regularly, while 93% of the study group and 90% of the control group reported consuming dinner. Lunch and afternoon snacks were less common, particularly in the psychiatric group.

Regularity of meal timing was lower among psychiatric patients: 50% reported always eating at regular times, 25% often, and 25% usually. In the control group, 50% reported regular meal timing often, and the remaining 50% usually. Regarding the timing of the last meal, 75% of the psychiatric group ate between 17:00 and 18:00, and 25% between 19:00 and 20:00, while 40% of control group participants ate after 21:00. Patients in the psychiatric group exhibited significantly lower levels of physical activity. Demotivating factors included lack of energy and limited access to exercise facilities.

#### 3.3.2. Stress and Eating Behavior

Stress influenced eating behaviors differently between groups. In the psychiatric group, 54% stated stress did not affect their eating, 32% reported loss of appetite during stress, and 14% engaged in compensatory overeating. In the control group, 60% reported no stress effect on eating, while 40% avoided food under stress.

Feelings of hunger were most frequently reported in the afternoon (46% in the study group, 40% in controls), followed by before noon (29% vs. 30%) and morning (25% vs. 20%). In both groups, the most common hunger-coping strategy was to eat “whatever is available” (86% psychiatric group; 80% control), while a small number reported drinking water or eating sandwiches.

Snacking between meals was common in both groups: 89% in the study group and 90% in the control group reported doing so occasionally. Snacks included sandwiches (36% in the study group vs. 30% in controls), pastries (25% vs. 40%), sweets, and salty snacks. Strong associations were noted between snacking habits and psychiatric status (Cramér’s V = 0.591).

#### 3.3.3. Food Choices and Preferences

Taste was the primary factor influencing food choices in both groups (100%). Appearance influenced 14% of the psychiatric group and 10% of controls. Peer influence affected 7% of the psychiatric group.

When assessing taste preferences, 32% of psychiatric patients and 30% of controls preferred salty foods. Sour flavors were chosen by 29% in the study group and 40% in the control group. A preference for sweets was declared by 21% of the psychiatric group and 20% of controls, while fatty foods were preferred by 18% and 10%, respectively.

#### 3.3.4. Eating Habits and Dining out

Most psychiatric patients (71%) did not monitor caloric intake, compared to 40% in the control group. Only 18% of patients occasionally monitored calories depending on the product, similar to 30% of controls.

In terms of dining out, 43% of psychiatric patients and 60% of controls used fast food services. Restaurants were used by 18% of psychiatric patients and 40% of controls, while cafés were less common. A total of 36% of psychiatric patients and none of the control group reported not using foodservice facilities. Fast food consumption once a week was reported by 32% of psychiatric patients and 50% of controls.

#### 3.3.5. Breakfast and Staple Food Consumption

The most common breakfast choices among psychiatric patients were sandwiches (50%) and dairy products (46%). In the control group, 90% consumed sandwiches and 10% dairy. Cereal products were consumed daily by 93% of the psychiatric group and 70% of the control group. All participants reported using butter as a bread spread.

#### 3.3.6. Main Meal Composition and Protein Sources

Two-course dinners were consumed by 82% of psychiatric patients and 60% of controls. Red meat and poultry were consumed equally in the psychiatric group (50% each), whereas in the control group, red meat (70%) was more prevalent than poultry (30%).

Fish consumption once a week was common (68% psychiatric group; 60% control). However, 14% of psychiatric patients and 30% of controls did not consume fish at all. Psychiatric patients preferred freshwater fish (54%), while controls favored sea fish (70%).

Egg-based dishes (e.g., scrambled eggs, pancakes) were consumed 2–3 times weekly by 75% of psychiatric patients and 60% of controls.

#### 3.3.7. Fruits, Vegetables, and Dairy Intake

Daily vegetable intake was reported by 96% of the psychiatric group and 80% of the control group. Fresh vegetables were preferred in both groups (100% psychiatric, 70% control). Cooked vegetables were reported only by 30% of the control group.

Fruit consumption once daily was reported by 68% of the psychiatric group and 90% of controls, while 25% of psychiatric patients did not eat fruit at all.

Dairy product consumption once daily was declared by 75% of psychiatric patients and 10% of the control group. Most psychiatric patients (71%) consumed whole milk, while 90% of the control group did not consume milk. Preferred dairy products included fruit yogurts (36%), natural yogurts (21%), and kefir (14%) in the study group.

#### 3.3.8. Fluids, Sweets, and Nuts

Among psychiatric patients, 61% consumed 5–6 glasses of fluids daily and 32% more than 8 glasses. In the control group, all consumed eight or more glasses daily. The most consumed beverages in the psychiatric group were tea (96%), mineral water (86%), and coffee (79%), while the control group preferred mineral water (100%) and coffee (90%).

Sweets were consumed 2–4 times weekly by 50% of the psychiatric group and 30% of the controls. Daily sweet consumption was reported by 40% of controls. Nut consumption was declared by 50% of the psychiatric group and 90% of controls.

Generally, the impact of dietary habits on the quality of life was analyzed among patients with mental disorders (study group) and healthy individuals (control group) (Table 3 and Table S1). The nutritional status was significantly influenced by factors such as the number of meals consumed per day, the type of meals, the presence of stress during meals, the timing of hunger sensations, methods of coping with hunger, the frequency of snacking between meals, factors influencing food choices, the type of breakfast consumed, the frequency of consuming cereal products, the number of courses for the main meal of the day, and the frequency of consuming fish, egg-based dishes, vegetables, fruits, and food types in general. On the other hand, factors such as meal regularity, timing of last meal, type of snacks, calorie intake monitoring, use of meal catering services, frequency of fast food consumption, type of bread spreads, types of meat, fish, fluids consumed, amount of fluids consumed, frequency of dairy product consumption, sweets, type of milk and dairy products, and nut consumption did not significantly impact nutritional quality between the study and the control groups. In addition to statistical significance (*p*-values), the strength of associations between categorical dietary variables and group affiliation (psychiatric vs. control) was assessed using Cramér’s V coefficient. Strong associations (V ≥ 0.5) were identified for *type of meals during the day* (V = 0.590), *impact of stress during meals* (V = 0.525), *type of snacks* (V = 0.591), and *factors influencing food choice* (V = 0.635). Moderate associations (V = 0.3–0.49) were observed for the *number of meals per day* (V = 0.405), *meal regularity* (V = 0.488), and *frequency of cereal and vegetable consumption* (V = 0.520 and V = 0.520, respectively), among others. These results suggest that specific dietary behaviors exhibit varying degrees of relationship with psychiatric status.

### 3.4. Use of Physical Activity Patterns and Stimulants

Although no statistically significant differences were found in stimulant use between the groups (*p* > 0.05), a noticeable trend toward higher alcohol and tobacco use was observed in the psychiatric group.

In our study, we mean physical activity according to the WHO definition. The World Health Organization (WHO) defines physical activity as body movement caused by skeletal muscles, which requires energy input [24].

Engagement in any form of physical activity was reported by 82% of psychiatric patients and 80% of control participants. Improvement of the physical appearance constituted the main motivating factor for physical activity, both in the study group (75%) and in the control group (80%). Health-related motivation, such as maintaining a healthy body weight, was declared by 57% of the study group and 20% of the control group. Additionally, the desire to remain in good shape was declared by 4% of patients in the study group. The frequency of engagement in physical activity once a week was declared by 64% of patients in the study group and by 60% of participants in the control group. Physical activity practiced twice a week was reported by 25% of the study group and 20% of the control group. Notably, physical activity undertaken three times or four times a week was declared by 4% of patients from the study group in each case. In the control group, physical activity practiced both three and four times a week was reported by 10% of participants, respectively.

In the present study, the intensity of physical activity was specified in quantitative terms. Interestingly, 75% of patients in the study group and 30% of participants in the control group did not perform intensive exercises. On the other hand, 60% of the control group and 54% of the study group practiced intensive sports once a week. Intensive exercises 2–3 times a week were declared by 7% of the study group and 10% of the control group.

The perception of the health benefits of physical activity was reported by 96% of participants in the study group and 40% of the control group. However, benefits related to improved body shape were recognized by 39% of the study group and 60% of the control group. Factors hindering physical activity in both groups were also analyzed, revealing two main reasons: (1) reluctance—cited by 79% of the study group and 20% of the control group—and (2) lack of time—indicated by 50% of the study group and as much as 70% of the control group. Additionally, poor health was mentioned by 7% of the patients in the study group and 10% of the control group.

In the context of the characteristics and frequency of physical activity, a significant overall effect on the health status of patients was observed (Table 4). In particular, motivation, frequency of physical activity, frequency of intensity of effort, perceived benefits of practicing sports, and factors hindering physical activity had a significant impact on the quality of life between the two study groups. In contrast, the simple declaration of being physically active did not show a significant effect on the patients’ health status. In the domain of physical activity, differences between the clinical and control groups were further explored using Cramér’s V to assess the strength of association between categorical variables and psychiatric status. The strongest association was found for the frequency of intense physical exercise (Cramér’s V = 0.657), suggesting a distinct divergence in high-effort physical activity engagement between the groups. Moderate associations were observed for motivation for physical activity (V = 0.527), with individuals from the control group more often citing weight control and esthetic reasons, while psychiatric patients less frequently declared any conscious motivational drive. The frequency of general physical activity also showed a moderate relationship with group affiliation (V = 0.425), as the clinical group reported less frequent engagement in regular physical activity. Additional moderate associations were found for benefits attributed to sport (V = 0.456), where healthy participants more often identified health- and body-related advantages, and for barriers to physical activity (V = 0.489), with reluctance and lack of time being more prevalent among the clinical group. These results indicate clear discrepancies in physical activity behaviors and attitudes depending on psychiatric status.

By contrast, the types of stimulants used did not have a significant impact on the quality of life in either group of subjects (Table 5).

The present study examined the frequency and patterns of stimulant use. Among patients in the study group, 75% did not consume alcohol, 14% consumed it occasionally, and 11% reported drinking once a week. Similarly, in the control group, 50% consumed alcohol once a week, 30% occasionally, and 20% several times a week. As for smoking, 25% of psychiatric patients reported smoking, while none of the control group participants did. In contrast, 100% of participants in the control group reported that they did not smoke. Information concerning narcotics use was also obtained, and all participants in both groups declared non-use. In the domain of stimulant use, a strong association was found between group affiliation and the frequency of alcohol consumption (Cramér’s V = 0.526), indicating considerable differences in drinking habits between psychiatric patients and the control group. In contrast, no associations were observed for smoking or drug use (Cramér’s V = 0.000 for both), reflecting minimal variability or uniformly low prevalence in the studied groups.

## 4. Discussion

The findings of this study highlight the significance of specific lifestyle-related behaviors—particularly in the domains of nutrition, physical activity, and health awareness—among psychiatric inpatients. These behaviors directly impact cognitive and functional status and may affect the course of mental illness and the patient’s quality of life. Notably, significant differences were found between the study and control groups in various health behaviors and subjective assessments of well-being. The observed disparities are consistent with previous research emphasizing the vulnerability of individuals with psychiatric disorders to poor diet quality, limited health literacy, and sedentary lifestyles.

The concept of metabolic programming underscores how cumulative health behaviors can influence long-term outcomes. Our results support this notion by revealing behavioral patterns that may predispose psychiatric patients to chronic conditions. In the present study, differences between the psychiatric and control groups were particularly evident in subjective material status, dietary patterns, media sources of health information, and self-assessed health condition. Interestingly, sleep duration and subjective evaluation of dietary habits did not differ significantly, potentially reflecting limited self-awareness or denial of poor dietary choices among hospitalized patients—an observation previously reported in psychiatric nutrition literature.

Fojcik et al. [8] have emphasized the role of education in supporting patients with schizophrenia, noting that integrated educational programs improve health awareness and behavioral engagement. Similar approaches may be beneficial for patients with chronic psychiatric conditions. In line with these findings, our study indicates the need for targeted educational interventions to promote healthy lifestyle behaviors and to reduce the recurrence of disease symptoms.

Nutrition has significant differences with psychiatric status, particularly in terms of meal frequency, meal composition, reactions to stress, hunger management strategies, snacking, and food choice drivers. The inclusion of Cramér’s V coefficients provided additional insight into the strength of these associations, with the strongest links observed for stress-related eating, snacking habits, and reliance on taste over health-related factors in food selection. These findings suggest that dietary behavior in psychiatric patients is influenced not only by environmental factors but also by underlying psychopathological mechanisms, such as impulsivity or emotional dysregulation.

Consistent with literature reports, the Western-style diet—characterized by high intake of saturated fats, trans fats, refined carbohydrates, and sodium—is associated with nutrient deficiencies and inflammation, both of which may exacerbate psychiatric symptoms. In our previous study on the same patient population, we identified deficiencies in vitamin D and vitamin B12 [28,29,30,31], which are essential for neuroimmune regulation. Likewise, limited intake of polyunsaturated fatty acids (PUFAs), typically found in fish, nuts, and seeds, may compromise neuroprotection and cognitive function [32,33,34,35]. Excessive intake of arachidonic acid from red meat and overconsumption of salt- and sugar-sweetened products can stimulate pro-inflammatory pathways, further worsening mental and metabolic health [10,36].

Although this study did not find significant group differences in probiotic consumption or gut-supporting dietary patterns, the literature evidence increasingly supports the gut–brain axis as a crucial factor in mental health [37,38,39]. Disruption of the intestinal microbiota and chronic inflammation are linked to the development of neuropsychiatric symptoms. Therefore, promoting diets rich in fiber, prebiotics, and probiotics remains a valuable strategy in psychiatric care.

In terms of physical activity, strong and moderate associations with psychiatric status were observed across multiple variables, including frequency and intensity of exercise, perceived benefits, and barriers to activity. Psychiatric inpatients reported significantly lower physical activity levels, citing reluctance and lack of motivation more frequently than controls. Although general declarations of being physically active did not differ significantly, detailed analysis showed that patients were less likely to engage in regular or high-intensity exercise. This is a critical finding, as physical inactivity has been linked to increased risk of cardiovascular and metabolic disorders, which in turn can exacerbate psychiatric symptoms [40].

The role of stimulant use (alcohol, tobacco, drugs) was also examined. While differences in overall consumption were not statistically significant, a strong association was found for alcohol use, with psychiatric patients more frequently reporting occasional or weekly consumption. Smoking and drug use were rarely declared in either group, potentially due to institutional restrictions or underreporting. These findings align with literature suggesting that alcohol consumption is a common coping mechanism in psychiatric populations, underscoring the importance of including substance use monitoring in clinical assessments.

In addition, our results are in line with broader evidence supporting the use of dietary interventions to support cognitive health. Research indicates that adherence to Mediterranean and Nordic diets—rich in fruits, vegetables, whole grains, legumes, and healthy fats—is associated with improved cognitive performance and reduced dementia risk. A systematic review by Christodoulou et al. [41] demonstrated that both diets are beneficial for cognitive outcomes, with high adherence linked to delayed cognitive decline. Conversely, overconsumption of red meat and processed foods may have adverse effects, although some studies noted unexpected associations between red meat intake and improved cognition, likely reflecting complex cultural or contextual factors.

Given the progressive nature of cognitive impairment in disorders such as dementia, preventive strategies that incorporate nutritional guidance and physical activity are of growing importance. McMaster et al. [42] found that even short-term, structured lifestyle interventions—including modules on diet, exercise, and cognitive training—can yield cognitive benefits in at-risk populations. The integration of such programs into psychiatric care models could offer a cost-effective and scalable approach to improving outcomes.

One of the key limitations of our study is the reliance on self-reported data, which may be influenced by cognitive dysfunction, particularly in dementia patients. To mitigate this, psychiatric supervision was ensured during data collection, and only patients deemed capable of meaningful participation were included. Additionally, while the sample size was relatively small, the findings contribute valuable insight into an understudied patient group.

Overall, the results emphasize the need for comprehensive, multidisciplinary interventions that address the nutritional and lifestyle needs of psychiatric inpatients. Future programs should incorporate education tailored to cognitive abilities, promote access to healthy foods, and encourage safe and feasible physical activity. Although causality cannot be inferred, the associations observed here suggest that improving diet and activity levels could enhance not only physical health but also mental resilience and quality of life in psychiatric populations.

## 5. Limitations of the Study

This study has several important limitations. First, the reliability of self-reported data in the clinical group may be compromised due to cognitive deficits commonly observed in patients with chronic psychiatric conditions, including dementia. Although only cognitively competent individuals were selected for participation, some inaccuracies may have persisted. Second, there was a notable demographic mismatch between the study and control groups—patients were generally older and had lower educational attainment, while controls were younger and more educated. These variables could independently affect health behaviors and perceptions, complicating the isolation of psychiatric status as the primary factor.

Furthermore, the cross-sectional design of the study precludes causal inferences. Longitudinal studies are needed to monitor behavioral changes over time and determine the impact of targeted interventions. Another limitation is the hospital nutrition model itself, which is often standardized for metabolic disorders and may not sufficiently address micronutrient deficiencies relevant to mental health. Lastly, the sample size was relatively small, especially in the control group, which limits the generalizability of the results. Despite these limitations, the study offers important insights into modifiable health behaviors in psychiatric populations and lays the groundwork for future, more controlled research.

The use of ICD-10 rather than the updated ICD-11 is justified by the fact that ICD-10 was still in routine clinical use at the time of data collection (January–December 2022). Moreover, the psychiatric hospital had not yet implemented ICD-11 in its diagnostic procedures. This transitional gap in clinical practice may temporarily affect research consistency but does not invalidate the diagnostic validity under ICD-10 criteria. Although the study and control groups differed in age and education, this reflects real-world clinical conditions. Due to ethical and logistical constraints in matching, group selection prioritized diagnostic status, with psychiatric oversight ensuring valid responses. These differences are acknowledged as potential confounders.

## 6. Conclusions

This study provides preliminary insights into the health-related behaviors of long-term psychiatric inpatients. Observed dietary patterns—such as irregular meals, inadequate intake of essential nutrients, and stress-induced eating—were strongly associated with group affiliation. However, due to demographic mismatches between the psychiatric and control groups, it cannot be conclusively determined whether these differences stem from the psychiatric conditions themselves, underlying sociodemographic disparities, or both. Consequently, the findings should be viewed as hypothesis-generating rather than confirmatory. Nonetheless, the results point to the potential clinical value of incorporating structured dietary assessment and individualized nutrition support into psychiatric care. Future studies using demographically matched samples and larger cohorts are needed to clarify causal relationships and inform effective intervention strategies.

## Figures and Tables

**Table 1:** Characteristics of the sample.

Variables		*n* * = 28	*n* * = 10	*p* *
Gender	Women	10	3	0.034
	Men	18	7	
Age	18–25	0	3	
	26–35	0	0	
	36–46	8	4	
	47–57	3	2	
	58–68	15	1	
	≥69	2	0	
BMI (kg/m^2^)	<18.5	0	0	
	18.5–24.9	19	5	
	25.0–29.9	9	4	
	≥30	0	1	
Place of residence	City > 100,000 inhabitants	10	8	
	City < 100,000 inhabitants	12	2	
	Village	6	0	
Education	Primary	9	0	
	Vocational	9	1	
	High school	2	3	
	University	6	6	

BMI = body mass index; *n* * = number of participants or number of patients in the control group, *p* * = chi-squared test for all tested parameters.

**Table 2:** Selected quality of life characteristics.

Characterization		*n* = 28	*n* = 10	*p* *	*p* **
Material’s condition	Very good	3	2	0.006	0.004
	Good	6	7		
	Neither poor or good	10	1		
	Poor	8	0		
	Very poor	1	0		
Health condition	Very good	0	2	<0.001	
	Good	14	7		
	Poor	13	1		
	Very poor	1	0		
The way of nutrition	Very good	0	2	<0.001	
	Good	26	8		
	Poor	2	0		
	Very poor	0	0		
Number of hours of sleep	<7 h	8	1	0.0437	
	7–8 h	15	8		
	>8 h	5	1		
Evaluation of the source of information about a healthy lifestyle	Television, radio	15	2	0.037	
	Internet	6	8		
	Books, magazines	6	0		
	School	0	0		
	Family	1	0		

*p* *, chi-squared test for individual characteristics examined; *p* **, chi-squared test for all characteristics examined.

**Table 3:** Nutrition with significant differences.

Characterization		*n* = 28	*n* = 10	*p* *
Number of meals per day	1	0	0	0.004
	2	1	0	
	3	24	7	
	4	3	2	
	5	0	1	
Type of meals during the day	Breakfast	28	10	0.008
	Lunch	2	4	
	Diner	26	9	
	Afternoon tea	2	1	
	Supper	28	10	
The impact of stress during meals	yes, I eat more then	0	0	0.040
	yes, I eat like crazy	4	0	
	I do not eat at all then	9	4	
	Stress does not affect my diet	15	6	
Times of feeling hungry	In the morning	7	2	0.036
	Before noon	8	3	
	In the afternoon	13	4	
	In the evening	0	1	
A way of satisfying hunger	I eat anything	24	8	0.001
	I drink water	1	0	
	I eat sweets	0	1	
	I eat dairy products	1	0	
	I eat sandwiches	2	1	
	I am holding back	1	0	
Frequency of snacking between meals	Yes, always	0	0	0.001
	Yes, often	3	1	
	Yes, sometimes	25	9	
	No, never	0	0	
Factors influencing the choice of food products	Taste	28	10	0.001
	Appearance	4	1	
	Family	0	0	
	Colleagues	2	0	
	Advertising	0	0	
	Knowledge about health benefits	0	0	
	Other	0	0	
Meals for breakfast	I do not eat breakfast	0	0	0.050
	Dairy products	13	1	
	Sandwiches	14	9	
	Vegetables	1	0	
	Fruit	0	0	
	Eggs	0	0	
	Others	0	0	
Frequency of eating cereal products	Never	0	0	0.005
	Occasionally	0	1	
	2–3 times a week	0	0	
	Every day	26	7	
	More than once a day	2	2	
Number of dishes during diner	I do not eat dinner	2	1	0.002
	One: only soup	1	1	
	One: only the second course	2	2	
	Two	23	6	
	Three	0	0	
Frequency of eating fish	I do not eat fish	4	3	0.018
	1 time a week	19	6	
	2 times a week	5	1	
	3 times a week	0	0	
	4 times a week or more	0	0	
Frequency of eating dishes made from chicken eggs (boiled eggs, scrambled eggs, omelet, pancakes)	I do not eat eggs	0	0	0.017
	1 time a week	2	0	
	2–3 times a week	21	6	
	4 times a week	5	3	
	everyday	0	1	
Frequency of eating vegetables	I do not eat vegetables	0	0	0.004
	1 time a day	27	8	
	2 times a day	1	2	
	3 times a day	0	0	
	4 times a day or more	0	0	
Form of vegetables consumed	Fresh	28	7	0.034
	Cooked	0	3	
	Frozen	0	0	
	Dried	0	0	
	From a can	0	0	
Frequency of eating fruit	I do not eat fruit	7	0	0.024
	1 time a day	19	9	
	2 times a day	2	1	
	3 times a day and more	0	0	
Type of food eaten	Salty	9	3	0.036
	Sour	8	4	
	Sweets	6	2	
	Fatty	5	1	
Type of dairy products consumed	I do not eat dairy products	6	6	0.050
	Natural yogurt	6	2	
	Fruit yogurt	10	1	
	Kefir	4	1	
	Buttermilk	2	0	

*p* *, chi-squared test.

**Table 4:** Physical activity characteristics and frequency of engagement.

Characterization of Physical Activity		*n* = 28	*n* = 10	*p* *	*p* **
physical activity	yes	23	8	0.128	<0.001
	no	15	1		
motivation for physical activity	the desire to improve external appearance	21	8	0.024	
	the desire to maintain proper body weight	16	2		
	the desire to maintain good physical condition	1	0		
	the desire to improve well-being	0	0		
	willingness to follow fashion	0	0		
	the desire to be healthy	0	0		
	others	0	0		
frequency of physical activity	I do not engage in physical activity	11	1	0.007	
	1 time a week	18	6		
	2 times a week	7	2		
	3 times a week	1	0		
	4 times a week	1	1		
	5 times a week	0	0		
	everyday	0	0		
frequency of intense physical exercise	I do not do intense physical exercise	21	3	0.033	
	1 time a week	15	6		
	2–3 times a week	2	1		
	4 times a week or more	0	0		
Benefits of practicing sports	health	27	4	0.049	
	better figure	11	6		
	better physical activity	0	0		
	feeling better	0	0		
	achieving results	0	0		
	others	0	0		
factors hindering physical activity	reluctance	22	2	0.032	
	lack of time	14	7		
	lack of equipment	0	0		
	lack of space	0	0		
	poor health	2	1		
	shame	0	0		
	others	0	0		

*p* *, chi-squared test for individual characteristics examined; *p* **, chi-squared test for all characteristics examined.

**Table 5:** Frequency of stimulant use in both groups.

Characterization of Stimulants		*n* = 28	*n* = 10	*p* *	*p* **
Frequency of drinking alcoholic beverages	several times a week	0	2	0.108	0.104
	once a week	3	5		
	occasionally	4	3		
	no	21	0		
Smoking	yes	7	0	0.121	
	no	21	10		
Drugs	yes	0	0	1.000	
	no	28	10		

*p* *, chi-squared test for individual characteristics examined; *p* **, chi-squared test for all characteristics examined.

## Data Availability

All data is available in the article.

## Supplementary Materials

The following supporting information can be downloaded at: https://www.mdpi.com/article/10.3390/nu17142315/s1, Table S1. Nutrition of no significant differences.
