# Supplementary material for: Assessment of Health-Related Behaviors in Patients Hospitalized with Chronic Psychiatric Disorders—A Case-Control Study from a Closed Psychiatric Ward
Source: Nutrients. 2025 Jul 14;17(14):2315. doi: 10.3390/nu17142315 (PMC12299660; doi:10.3390/nu17142315)
Supplement: Supplementary file 1 [file nutrients-17-02315-s001.zip › nutrients-3705169-supplementary.pdf]

nutrients

MDPI

Supplementary Materials

Table S1. Nutritions of no significant differences

Characterization		N=28	N=10	*p**
Meals eaten regularly	Always	14	0	1.000
	Often	7	5	
	Usually	7	5	
	Never	0	0	
time of the last meal	17-18	21	0	0.686
	19-20	7	6	
	after 21	0	4	
	irregularly, at different hours	0	0	
Type of snacks	sandwiches	10	3	0.487
	sweets	2	6	
	chips, sticks	1	7	
	vegetables	0	0	
	fruit	1	7	
	yoghurts	0	0	
	buns, sweet rolls	7	4	
	fast food products	1	1	
	nuts	0	0	
	others	0	0	
Controlling the calorie content of meals	always	0	0	0.265
	depends on the products	3	3	
	occasionally	5	3	
	I don't pay attention	20	4	
Use of collective catering establishments	yes, fast food	12	6	0.508
	yes, restaurant	5	4	
	yes, coffehouse	1	0	
	no	10	0	
Frequency of eating fast food	everyday	0	0	0.535
	every othe day	1	2	
	one a week	9	5	
	occasionally	8	3	
	no	10	0	
Type of bread spread	I don't spread bread	0	0	0.559
	butter	28	10	
	margarine	0	0	
	cholesterol-free margarine	0	0	
	others	0	0	

Type of meat	I don't eat meat	0	0	0.386
	red meat (beef, pork, lamb)	14	7	
	poultry meat (chicken, turkey, rabbit)	14	3	
Type of fish eaten	I don't eat fish	4	3	0.686
	sea fish (salmon, mackerel, herring, halibut, cod, tuna)	9	7	
	freshwater fish (trout, carp, pike)	15	0	
The amount of fluids drinks	2 glasses	0	0	0.675
	2-4 glasses	2	0	
	5-6 glasses	17	0	
	8 glasses and more	9	10	
Type of drinks consumed	Tea	27	7	0.126
	coffe	22	9	
	mineral water	24	10	
	flavored water	1	0	
	milk	8	0	
	juice	2	7	
Frequency of dairy products consumed	I don't eat dairy products	7	9	0.194
	1 times a day	21	1	
	2 times a day	0	0	
	3 times a day and more	0	0	
	4 times a day or more	0	0	
Type of milk consumed	I don't drink milk	7	9	0.133
	whole milk (3.2% fatt)	20	1	
	skimmed milk (1-2%)	1	0	
	fat-free milk	0	0	
	flavored milk	0	0	
Frequency of sweets consumed	I don't eat sweets	3	1	0.882
	1 time a week	11	2	
	2-4 times a week	14	3	
	everyday	0	4	
Nuts consumption	yes	14	9	1.000
	no	14	1	

*p**, test chi-squared

Questionnaire on Eating Habits and Physical Activity

I. General Information

1. Gender:

a) Female

b) Male

2. Age (in years):
a) 18–25
b) 26–35
c) 36–46
d) 47–57
e) 58–68
f) Over 69
3. Place of permanent residence:
a) Large city
b) Small town
c) Village
4. What is your education level?
a) Primary
b) Vocational
c) Secondary
d) Higher
5. How do you assess your family's financial situation?
a) Very good
b) Good
c) Average
d) Rather poor
e) Very poor
6. How do you assess your health status?
a) Very good
b) Good
c) Sufficient
d) Poor
7. How do you assess your eating habits?
a) Very good
b) Rather good
c) Rather poor
d) Poor
8. How much time per day do you spend sleeping?
a) Up to 6 hours
b) 7–8 hours
c) More than 8 hours
9. Where do you get information about a healthy lifestyle? (multiple answers allowed)
a) Television, radio
b) Internet
c) Books, magazines
d) School
e) Family

II. Nutrition

Please enter your height (in centimeters): ________________________

Please enter your weight (in kilograms): ________________________

10. How many meals do you eat per day?

a) One

b) Two

c) Three

d) Four

e) Five

11. Do you eat the following meals daily? (select as appropriate)

a) Breakfast

b) Second breakfast

c) Lunch

d) Afternoon snack

e) Dinner

12. Do you eat your meals regularly (at fixed times)?

a) Always

b) Often

c) Sometimes

d) Never

13. At what times do you eat the following meals?

Meal - Time:

Breakfast: __________

Second breakfast: __________

Lunch: __________

Afternoon snack: __________

Dinner: __________

14. What time do you eat your last meal of the day?

a) 5–6 PM

b) 7–8 PM

c) After 9 PM

d) Irregular, at different times

15. Does stress affect your eating habits?

a) Yes, I eat more

b) Yes, I eat uncontrollably

c) I do not eat at all

d) Stress does not affect my eating habits

16. When do you most often feel hungry?

a) In the morning

b) Before noon

c) In the afternoon

d) In the evening

17. How do you usually satisfy hunger? (multiple answers allowed)

a) I eat anything

b) I drink water
c) I eat sweets
d) I consume dairy products
e) I eat sandwiches
f) I refrain from eating
18. Do you snack between meals?
a) Yes, always
b) Yes, often
c) Yes, sometimes
d) Never
19. If yes, what products do you usually consume? (multiple answers allowed)
a) Sandwiches
b) Sweets
c) Chips, pretzels
d) Vegetables
e) Fruits
f) Yogurts
g) Pastries, sweet rolls
h) Fast food
i) Nuts
j) Others, what? ____________
20. What factors influence your food choices? (multiple answers allowed)
a) Taste
b) Appearance
c) Family
d) Friends
e) Advertising
f) Knowledge about health benefits
g) Others, what? ____________
21. Do you monitor the caloric content of the products you eat?
a) Always
b) Depends on the product
c) Occasionally
d) I don't pay attention
22. Do you use mass catering services?
a) Yes, fast food bar
b) Yes, restaurant
c) Yes, café
d) No, I don't
23. How often do you eat fast food products?
a) Every day
b) Every other day
c) Once a week

d) Occasionally
e) I don't eat them
24. What do you usually eat for breakfast?
a) I don't eat breakfast
b) Dairy products
c) Sandwiches
d) Vegetables
e) Fruits
f) Eggs
g) Others, what? ____________
25. How often do you eat grain products?
a) I don't eat such products
b) Occasionally
c) 2–3 times a week
d) Daily
e) More than once a day
26. What do you usually spread on your bread?
a) I don't spread anything
b) Butter
c) Margarine
d) Cholesterol-free margarine
e) Other, what? ____________
27. How many dishes make up your typical lunch?
a) I don't eat lunch
b) One: only soup
c) One: only main course
d) Two
e) Three
28. What type of meat do you prefer?
a) I don't eat meat
b) Red meat (beef, pork, lamb)
c) Poultry (chicken, turkey, rabbit)
29. How often do you eat fish?
a) I don't eat fish
b) Once a week
c) Twice a week
d) Three times a week
e) Four times a week or more
30. Which types of fish do you prefer?
a) I don't eat fish
b) Saltwater fish (salmon, mackerel, herring, halibut, cod, tuna)
c) Freshwater fish (trout, carp, pike)
31. How often do you eat egg-based dishes (boiled eggs, scrambled eggs, omelets, pancakes)?
a) I don't eat eggs

b) Once a week
c) 2–3 times a week
d) 4 times a week
e) Daily
32. How many times a day do you eat vegetables?
a) I don't eat vegetables
b) Once a day
c) Twice a day
d) Three times a day
e) Four times a day or more
33. In what form do you prefer vegetables?
a) Fresh
b) Cooked
c) Frozen
d) Dried
e) Canned
34. How many times a day do you eat fruit?
a) I don't eat fruit
b) Once a day
c) Twice a day
d) Three times a day or more
35. What type of dishes do you prefer? (multiple answers allowed)
a) Salty
b) Sour
c) Sweet
d) Fatty
36. How many glasses of fluids do you drink daily?
a) 2 glasses
b) 3–4 glasses
c) 5–6 glasses
d) 8 glasses or more
37. What drinks do you most often consume? (multiple answers allowed)
a) Tea
b) Coffee
c) Mineral water
d) Flavored water
e) Milk
f) Juice
38. How many times a day do you consume milk and its products?
a) I don't consume them
b) Once a day
c) Twice a day
d) Three times a day
e) Four times a day or more

39. What type of milk do you usually drink?
a) I don't drink milk
b) Whole milk (3.2%)
c) Reduced-fat milk (1–2%)
d) Fat-free milk
e) Flavored milk
40. Which dairy products do you prefer?
a) I don't consume dairy products
b) Natural yogurt
c) Fruit yogurt
d) Kefir
e) Buttermilk
41. How often do you eat sweets?
a) I don't eat sweets
b) Once a week
c) 3–4 times a week
d) Daily
42. Do you eat nuts (walnuts, hazelnuts, almonds)?
a) Yes
b) No
43. What are your motivations for engaging in physical activity?
a) Desire to improve external appearance
b) Desire to maintain proper body weight
c) Desire to maintain good physical condition
d) Desire to improve well-being
e) Desire to follow trends
f) Desire to be healthy
g) Other, please specify: ....

44. How would you describe the frequency of your physical activity?
a) I do not engage in physical activity
b) Once a week
c) Twice a week
d) Three times a week
e) Four times a week
f) Five times a week
g) Every day

45. How often do you perform intense physical exercises (where your effort is high—you feel tired and sweat)?
a) I do not perform intense physical exercises
b) Once a week

c) 2–3 times a week

d) Four times a week or more

46. What benefits do you see from engaging in physical activity?

a) Health benefits

b) Better physique

c) Improved physical performance

d) Better well-being

e) Achieving results

f) Other, please specify: ....

47. What factors hinder your physical activity?

a) Reluctance

b) Lack of time

c) Lack of equipment

d) Lack of space

e) Poor health

f) Shame

g) Other, please specify: ....

48. Do you consume alcoholic beverages?

a) Yes, several times a week (which ones?)

b) Yes, once a week

c) Yes, occasionally

d) No

49. Do you smoke cigarettes?

a) Yes

b) No

50. Have you ever had personal contact with drugs?

a) Yes

b) No
